# Supplementary material for: Dorsomorphin inhibits AMPK, upregulates Wnt and Foxo genes and promotes the activation of dormant follicles
Source: Commun Biol. 2024 Jun 20;7:747. doi: 10.1038/s42003-024-06418-9 (PMC11190264; doi:10.1038/s42003-024-06418-9)
Supplement: Supplementary file 2 — Supplementary Information [file 42003_2024_6418_MOESM2_ESM.pdf]

Supplementary Figure S1. PRKAA1 and PRKAA2 sequencing data and antibody validation.

a

Gene	Primordial follicles				Primary follicles			
	Oocytes		Primordial follicle		Oocytes		Primary follicle	
	FPKM mean	P value	FPKM mean	P value	FPKM mean	P value	FPKM mean	P value
PRKAA1	**3.801**	**0.035**	**5.396**	**0.020**	2.779	0.300	**3.118**	**0.164**
PRKAA2	**4.820**	**0.018**	**3.711**	**0.176**	0.452	0.422	1.538	0.316

*[1]RNA sequencing data from (ref Ernst et al 2017, 2018)
*[2]Genes with significant FPKM values (noted in bold)

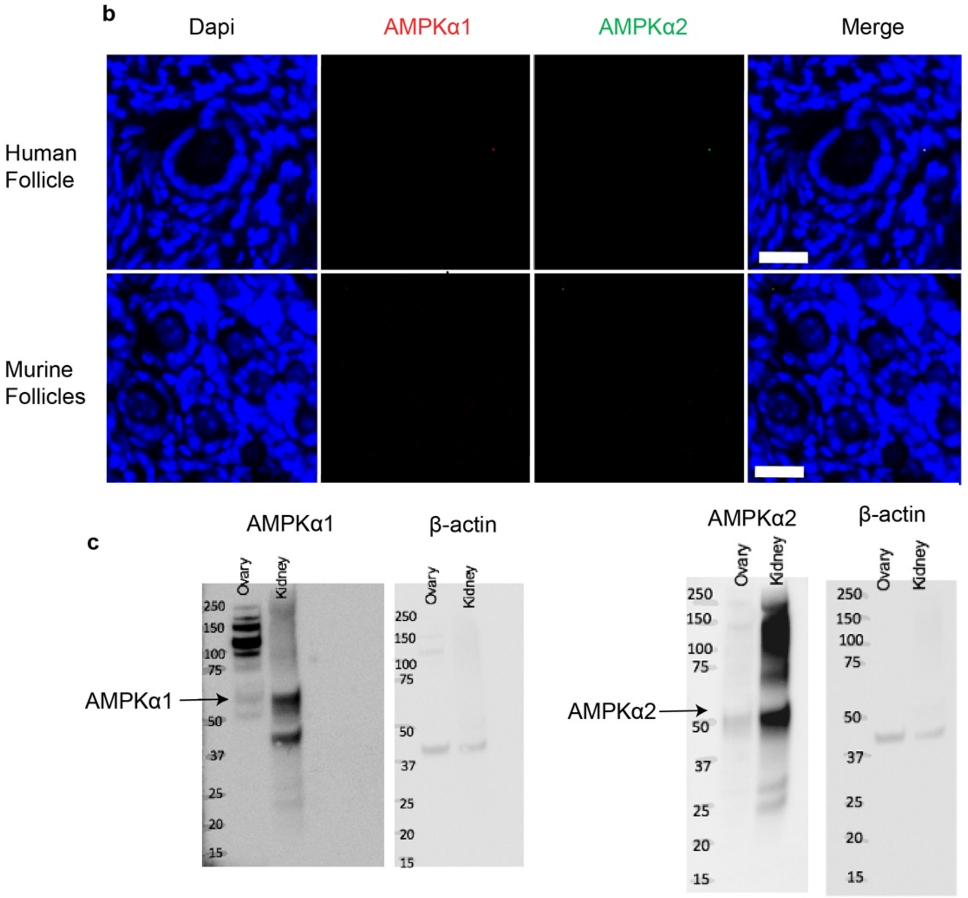

Supplementary Figure S1. PRKAA1 and PRKAA2 sequencing data and antibody validation. (**a**) FPKM values and p values are stated from stage-specific consistently expressed *PRKAA1* and *PRKAA2* genes in oocyte and granulosa cells, and significant p values are noted in bold. (**b**) Negative immunofluorescence controls omitting the primary antibodies against AMPKα1 and AMPKα2 and counterstained with DAPI to detect dsDNA. Scale bar: 10 µm. (**c**) Validation of the primary antibodies against AMPKα1 and AMPKα2 using Western blotting; kidney lysate was included as a positive control.

Supplementary Figure S2. Representative photomicrographs of whole ovaries cultured in vitro and stained with haematoxylin and eosin at low magnification.

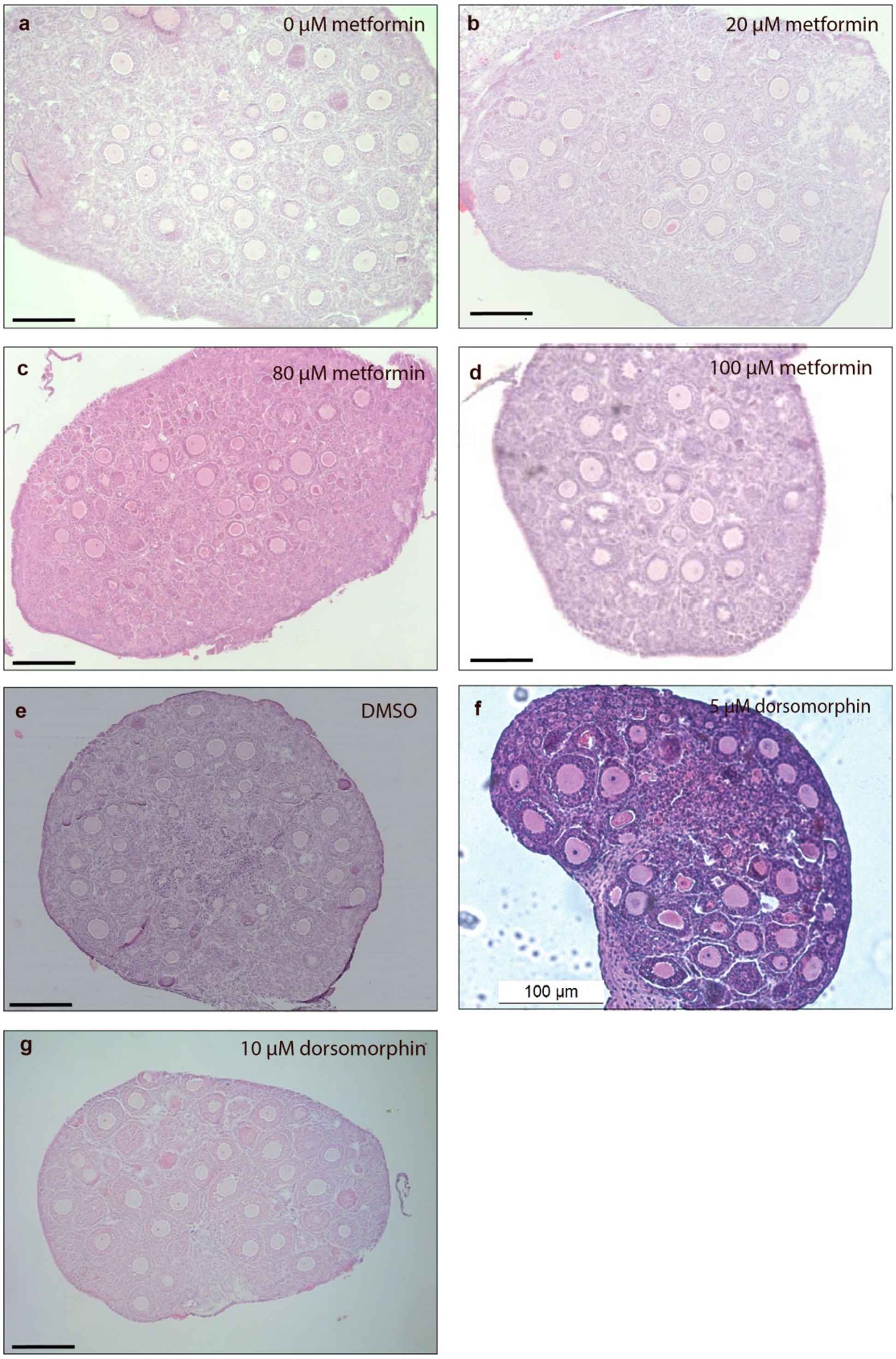

Supplementary Figure S2. Representative photomicrographs of whole ovaries cultured *in vitro* and stained with haematoxylin and eosin at low magnification. (**a**) 0 μM metformin. (**b**) 20 μM metformin. (**c**) 80 μM metformin. (**d**) 100 μM metformin. (**e**) DMSO-treated ovaries. (**f**) 5 μM dorsomorphin. (**g**) 10 μM dorsomorphin.

Supplementary Figure S3: Representative photomicrographs of whole ovaries cultured in vitro with BAY-3827 (AMPK inhibitor) and follicular distribution

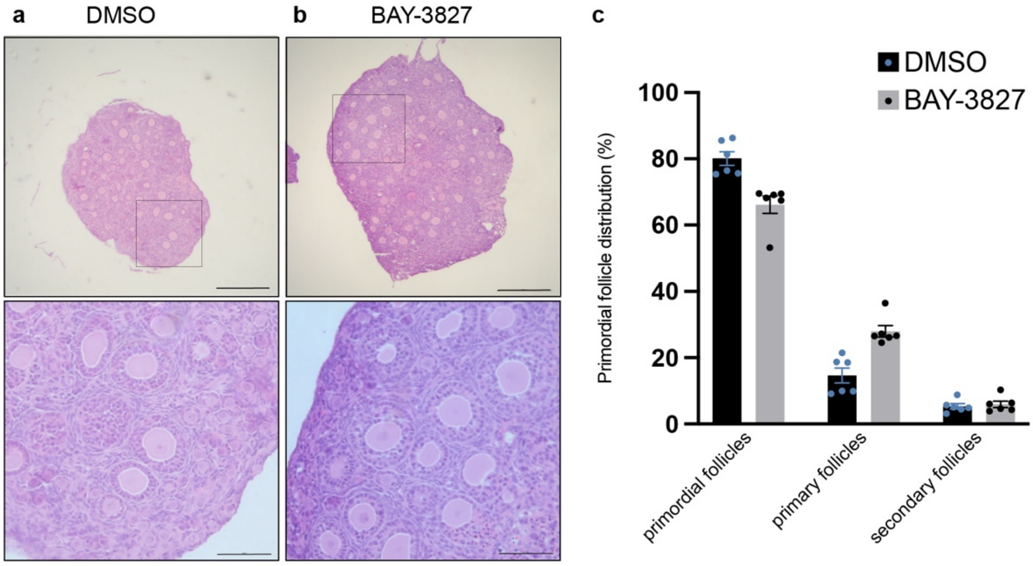

Supplementary Figure S3: Representative photomicrographs of whole ovaries cultured *in vitro* with BAY-3827 (AMPK inhibitor) and follicular distribution. (**a&b**) Ovaries under high and low magnification treated with DMSO (**a**) BAY-3827 (**b**). **(c)** Bar chart illustrating the follicular distribution in ovaries exposed to DMSO or BAY-3827, DMSO *n=6* biologically independent samples (ovaries) and BAY-3827 *n=6* biologically independent samples (ovaries). An unpaired t-test was performed. *P<0.05, **P<0.01, ***P<0.001, ****P<0.000. Scale bar in the upper row: 200 μm, and scale bar in the lower row: 50 μm.

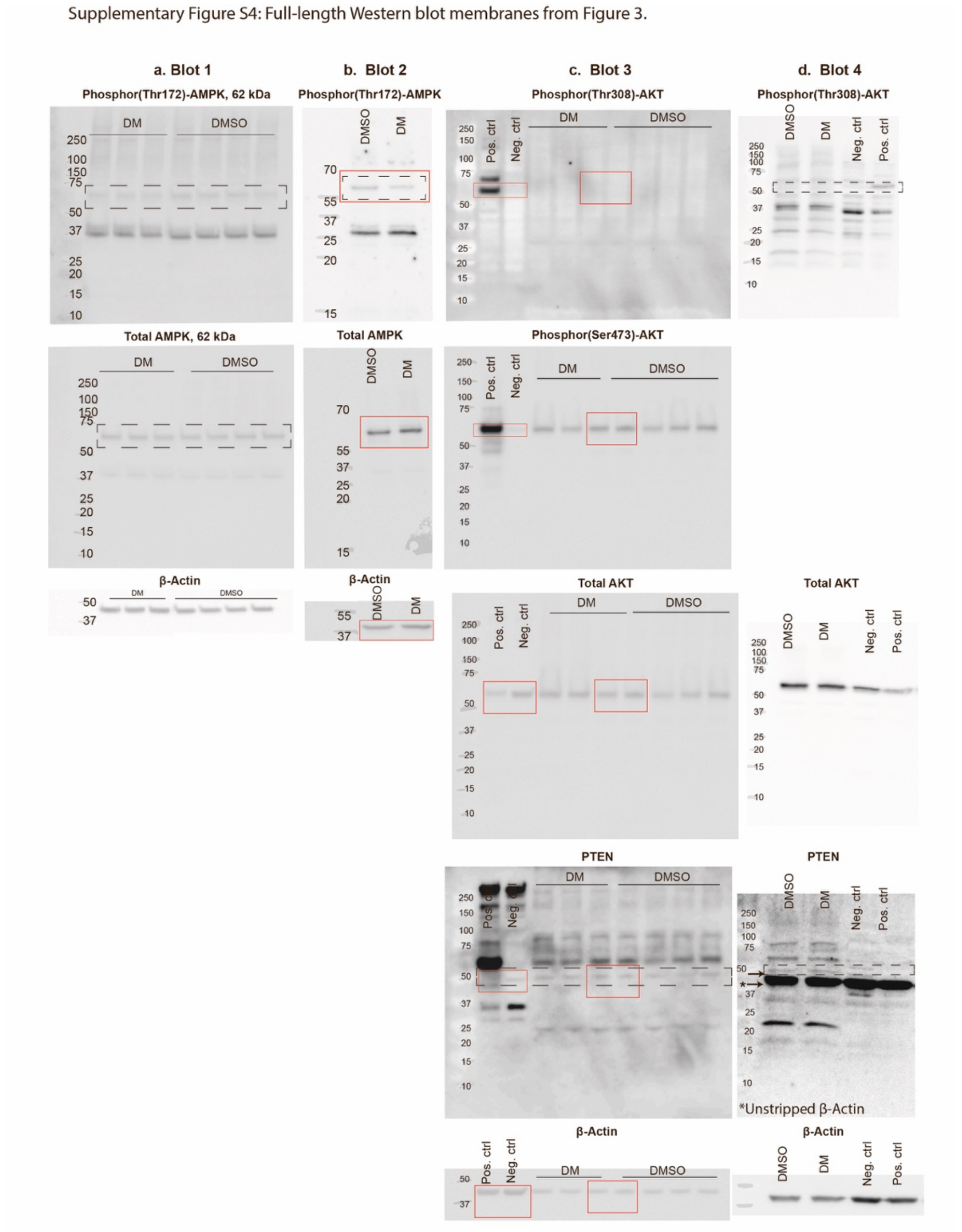

Supplementary Figure S4: Full-length Western blot membranes from Figure 3.
(**a&b**) Blots 1 and 2 were incubated with antibodies against phospho(Thr172)-AMPK and stripped before they were reprobed with anti-AMPK. Before reprobing, stripping control was conducted (not shown). Beta-actin was used as a loading control. (**c&d**) Blots 3 and 4 show full-length membranes incubated with anti-phospho(Thr308)-AKT, phospho(Ser4723)-AKT, total AKT, PTEN and β-actin as loading controls. If more than one band is visualized, a dotted square is made around the correct band. The red square marks the bands used in Figure 3.

Supplementary Figure S5: Cytoplasmic translocation of FOXO3A and p27 during follicle activation in in vitro cultured ovaries

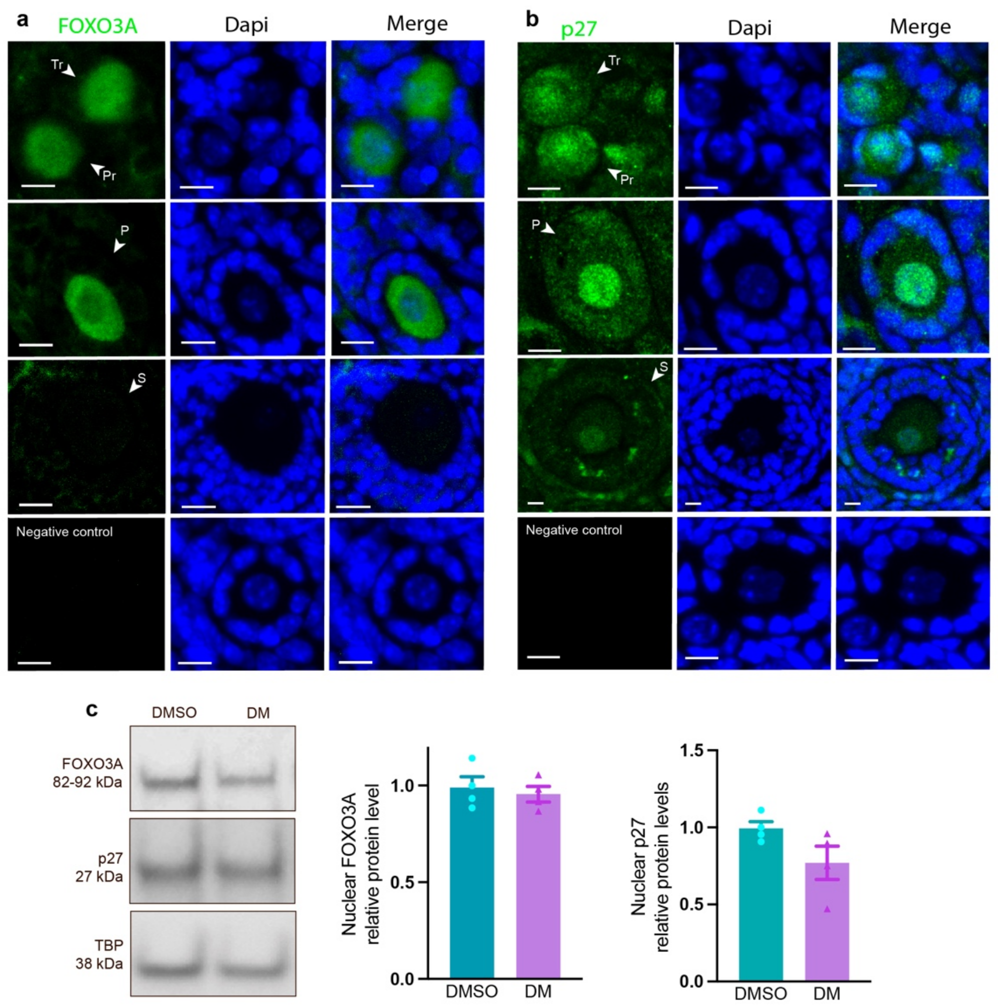

Supplementary Figure S5: Cytoplasmic translocation of FOXO3A and p27 during follicle activation in *in vitro* cultured ovaries. (**a**) Fluorescence microscopy images showing the distribution of FOXO3A in primordial (Pr), transitioning (Tr), primary (P) and secondary (S) follicles in ovaries cultured *in vitro* with DMSO *n=4* biologically independent samples (ovaries) consisting of 10-12 pooled ovaries/group or DM *n=4* biologically independent samples (ovaries) consisting of 10-12 pooled ovaries/group. The lower row shows a negative control where the primary antibody body against FOXO3A has been omitted. (**b**) Fluorescence microscopy images showing the distribution of p27 in primordial (Pr), transitioning (Tr), primary (P) and secondary (S) follicles in ovaries cultured *in vitro* with DMSO. Lower row: negative control. (**c**) Quantification of the nuclear fraction of FOXO3A and p27 using Western blotting and TBP as a nuclear loading control. Full-length Western blots are shown in **S6**. Scale bar: 20 µm.

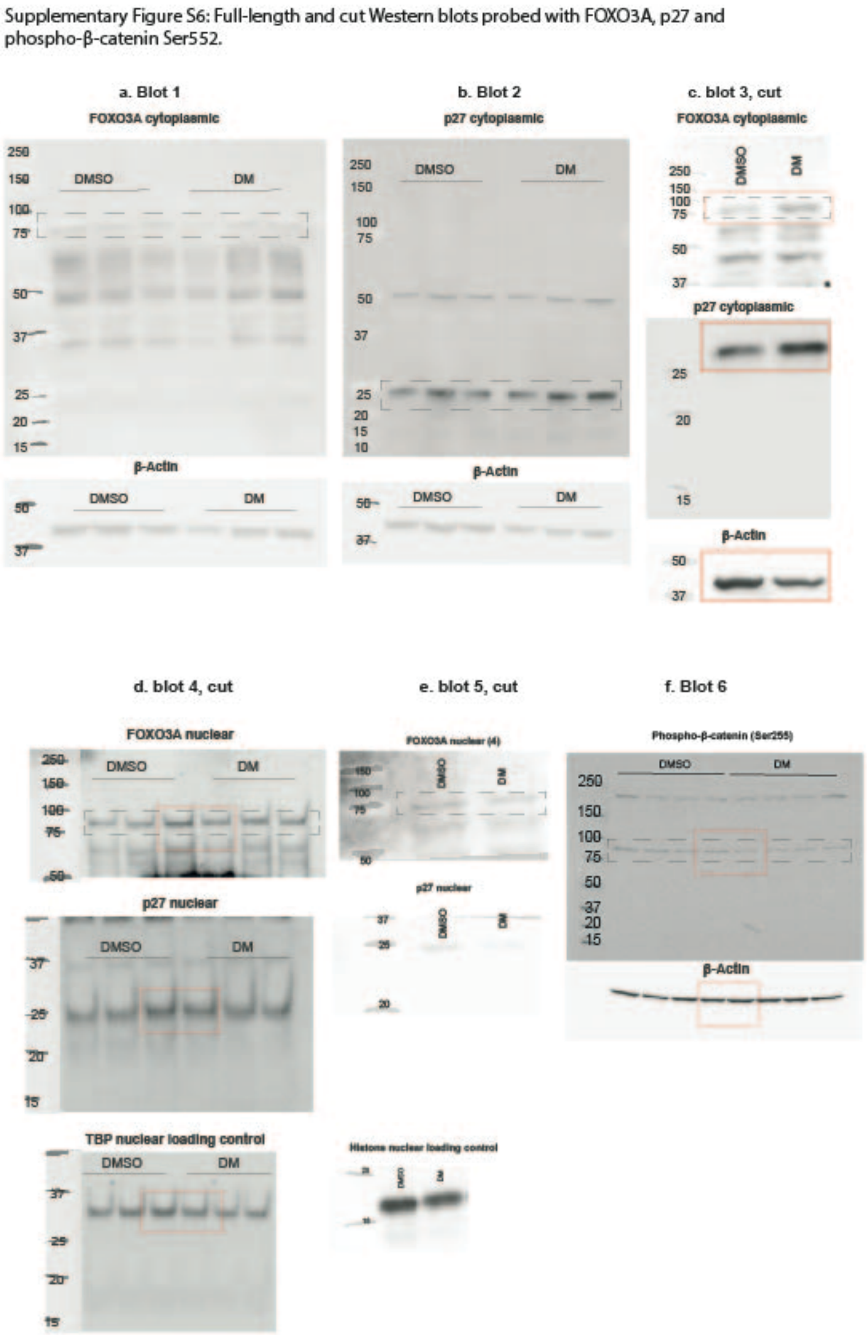

Supplementary Figure S6: Full-length and cut Western blots probed with FOXO3A, p27 and phospho-β-catenin Ser552.

(**a**) Blot 1 shows the full-length Western blotting of the cytoplasmic fraction probed with anti-FOXO3A and beta-actin as loading controls. The dotted square visualizes the band of interest. (**b**) Blot 2 illustrates the full-length blot of cytoplasmic fractions probed with anti-p27 and beta-actin as loading controls. (**c**) Membrane c is cut at ~ 37 kDa, and the top part is probed with anti-FOXO3A, whereas the lower part is incubated with anti-p27. Beta-actin was used as a loading control. (**d&e**)

Membranes are loaded with nuclear fractions and cut at ~ 50 or 37 kDa. The membranes were incubated with anti-FOXO3A and anti-p27 antibodies, and anti-TBP or anti-histone H3 antibodies were used as nuclear loading controls. (**f**) Full-length blot of whole-cell extracts from ovaries cultured for 6 hours with or without dorsomorphin and probed with an antibody against phospho-β-catenin Ser552. If more than one band is visualized, a dotted square is made around the correct band. The red square marks the bands used in Figures 4 and S5.

Supplementary Figure S7: Oocyte quality parameters

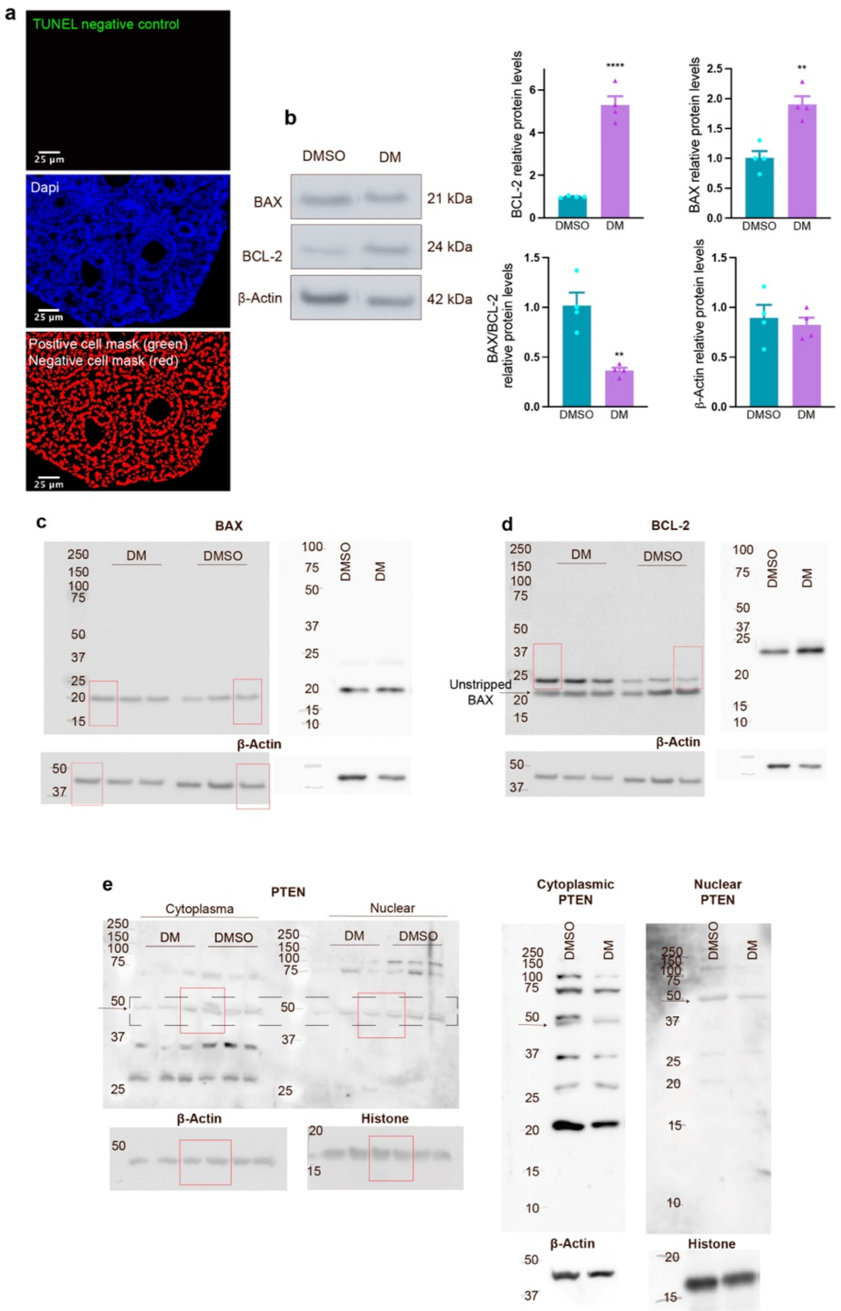

Supplementary Figure S7: Oocyte quality parameters

(**a**) Negative control omitting the reaction enzyme and counterstained with DAPI. (**b**) Quantification of BAX and BCL-2 in 5-day *in vitro* cultured ovaries using Western blotting. DMSO *n=4* biologically independent samples (ovaries) consisting of 4 pooled ovaries/group and DM *n=4* biologically independent samples (ovaries) consisting of 4 pooled ovaries/group . (**c-d**) Full-length Western blots

probed with anti-BAX and anti-BCL-2, and β-actin was used as a loading control. (**e**) Full-length Western blots loaded with nuclear and cytoplasmic fractions and probed with anti-PTEN. β-Actin was used as a loading control for the cytoplasmic fraction, and histone H3 was used as a loading control for the nuclear fractions.

Supplementary Table 1

Values provided as FPKM (Fragments Per Kilobase Million) for human data presented (Fig 1a) and as log2FoldChange (Extracted from: https://www.ncbi.nlm.nih.gov/geo/query/acc.cgi?acc=GSE230258) for mouse data (Figure 3b and 4a)

Gene	Oocytes from		Reference
	Primordial follicle	Primary follicle	
PRKAA1	3,80	2,78	Fig 1a
PRKAA2	4,82	0,45	
PPARGC1A	4,00	5,91	
ACACA	4,72	3,91	
Prkab1	3,68	-4,88	Fig 3b
Prkaa1	5,45	-5,32	
Stk11	0,21	-5,48	
Strada	-2,01	-2,60	
Cab39	-1,40	0,21	
Prkag2	-5,30	3,15	
Prkab2	-4,19	8,53	
Prkag1	-26,86	3,08	
Ppargc1a	2,86	16,57	
Acaca	4,10	4,81	
Wnt1	0	2,99	Fig 4a
Wnt2	0	-1,76	
Wnt2b	7,41	23,33	
Wnt4	1,62	9,05	
Wnt5a	-0,76	5,17	
Wnt5b	30,99	0	
Wnt6	-4,99	-5,23	
Wnt9a	-0,09	0	
Wnt10b	0	3,05	
Wnt11	11,22	0	
Ctnnb1	3,11	7,75	
Foxo1	-3,09	5,03	
Foxo3	0,65	1,11	
